# Supplementary material for: Central tropical Pacific convection drives extreme high temperatures and surface melt on the Larsen C Ice Shelf, Antarctic Peninsula
Source: Nat Commun. 2022 Jul 13;13:3906. doi: 10.1038/s41467-022-31119-4 (PMC9279480; doi:10.1038/s41467-022-31119-4)
Supplement: Supplementary file 1 — Supplementary Information [file 41467_2022_31119_MOESM1_ESM.pdf]

Supplementary Information for

Central tropical Pacific convection drives extreme high temperatures and surface melt on the Larsen C Ice Shelf, Antarctic Peninsula

Kyle R. Clem[1], Deniz Bozkurt[2,3,4], Daemon Kennett[1], John C. King[5], and John Turner[5]

[1] School of Geography, Environment and Earth Sciences, Victoria University of Wellington, Wellington, New Zealand
[2] Department of Meteorology, University of Valparaíso, Valparaíso, Chile
[3] Center for Climate and Resilience Research (CR)2, Santiago, Chile
[4] Center for Oceanographic Research COPAS COASTAL, Universidad de Concepción, Chile
[5] British Antarctic Survey, Natural Environment Research Council, Cambridge, UK

Contents of this file

Supplementary Figure 1 and Supplementary Figure 2

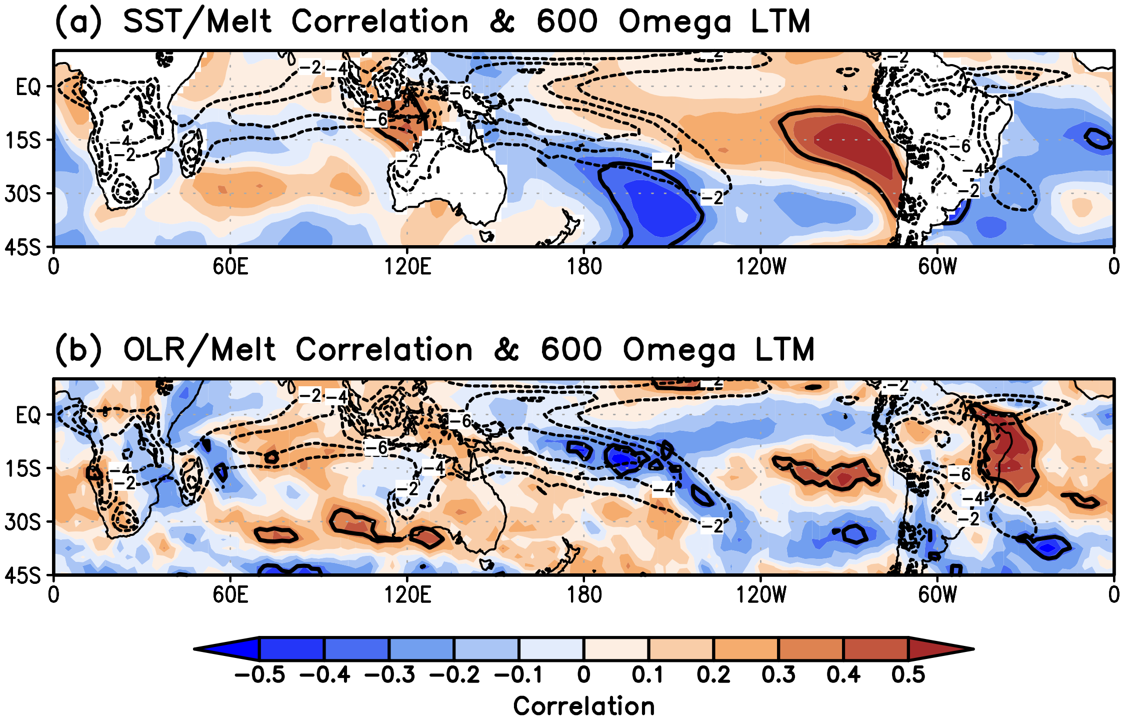

Supplementary Figure 1. The tropical regions favorable for generating a Rossby wave-induced teleconnection on the Larsen C ice shelf. The DJFM detrended correlation (shaded) and p<0.10 significance (bold contours) of Larsen C surface melt with (a) SST and (b) OLR, as in Figs. 1b and 1d, respectively, along with the 1979-2019 ERA5 climatological omega at 600 hPa (thin dashed contours; hPa s^{-1}). Only negative omega values (areas of climatological ascent) are shown, which denotes regions that are favorable for the development of deep tropical convection and upper-tropospheric divergent flow required to generate a Rossby wave[33].

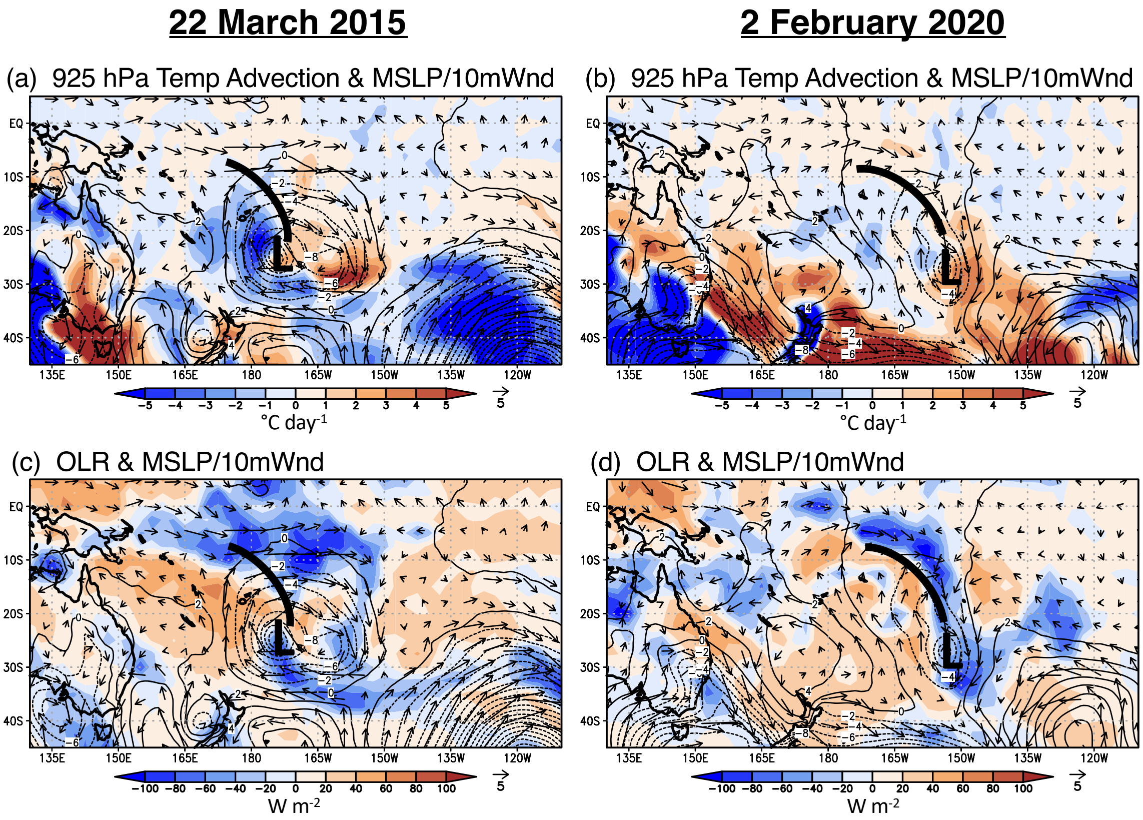

Supplementary Figure 2. The local synoptic conditions triggering CPAC convection preceding the two recent Antarctic Peninsula record-high temperatures. The (a-b) 925 hPa temperature advection (shaded) and (c-d) OLR (shaded) anomalies alongside MSLP (hPa, contours) and 10m wind (ms^{-1}, vectors) anomalies preceding the (left) 24 March 2015 and (right) 6 February 2020 record-high Antarctic Peninsula temperature events. The position of the surface low pressure center and its cold front are denoted by an "L" and curved black line, respectively.
